# Fiber-Optic Telecommunication Network Wells Monitoring by Phase-Sensitive Optical Time-Domain Reflectometer with Disturbance Recognition

**DOI:** 10.3390/s23104978

**Published:** 2023-05-22

**Authors:** Andrey A. Zhirnov, German Y. Chesnokov, Konstantin V. Stepanov, Tatyana V. Gritsenko, Roman I. Khan, Kirill I. Koshelev, Anton O. Chernutsky, Cesare Svelto, Alexey B. Pnev, Olga V. Valba

**Affiliations:** 1 Bauman Moscow State Technical University, 2-nd Baumanskaya 5-1, 105005 Moscow, Russia; stkv@bmstu.ru (K.V.S.); chobantv@yandex.ru (T.V.G.); khan.roman.igorevich@gmail.com (R.I.K.); koshelev-k@yandex.ru (K.I.K.); chernutsky.a@bmstu.ru (A.O.C.); pniov@bmstu.ru (A.B.P.); 2 Department of Applied Mathematics, MIEM, National Research University Higher School of Economics, 123458 Moscow, Russia; gchesnokov97@yandex.ru (G.Y.C.); valba@hse.ru (O.V.V.); 3 Dipartimento di Elettronica, Informazione e Bioingegneria, Politecnico di Milano, 20133 Milan, Italy; cesare.svelto@polimi.it; 4 Laboratory of Complex Networks, Brain and Consciousness Research Center, 119991 Moscow, Russia

**Keywords:** fiber optic sensor, distributed fiber optic sensor, phi-OTDR, acoustic monitoring, machine learning, telecommunication well

## Abstract

The paper presents the application of a phase-sensitive optical time-domain reflectometer (phi-OTDR) in the field of urban infrastructure monitoring. In particular, the branched structure of the urban network of telecommunication wells. The encountered tasks and difficulties are described. The possibilities of usage are substantiated, and the numerical values of the event quality classification algorithms applied to experimental data are calculated using machine learning methods. Among the considered methods, the best results were shown by convolutional neural networks, with a probability of correct classification as high as 98.55%.

## 1. Introduction

Fiber-optic technologies have been actively developing due to the growth of communication systems based on them since the 1980s [1,2,3,4,5,6]. The concomitant development of different technologies led to significant progress. It also occurred in related areas, one of which is fiber-optic sensors [7,8,9]. In the last decade, we have seen the development of new sensor types that can be implemented by exploiting already existing or specifically designed optical fibers: distributed fiber sensors [10,11,12,13,14]. They have a previously unattainable capability—interrogation of a few thousand points using a single cable without a power supply and without forming any special interrogation structures along the fiber line. This work is devoted to the combination of both of these capabilities when the fiber-optic network is complemented by an external monitoring system based on a phase-sensitive optical time-domain reflectometer (phi-OTDR): a fiber-optic distributed sensor.

Fiber-optic networks can be classified into trunk and urban networks according to the length of the section and branching [15]. Trunk lines are located between cities, have a linear length of tens of kilometers, a small number of intermediate branches, and are implemented on cables with a large number of fiber cores. City lines are located in urban infrastructure, typically using special collectors or pipes, and have a large number of branches passing through cable wells. These wells provide access to the junction points of fiber lines as well as other equipment and cables, using the same elements of urban infrastructure for installation.

The absence of copper cores in fiber cables makes them not only more compact and convenient but also less attractive for theft. There are practically no components for recycling and resale in them, unlike metals in wired cables. However, fiber lines need integrity monitoring. The main dangers for them are the following: Firstly, they may be confused with copper cables during theft. Secondly, they may be damaged accidentally during construction or repair work near the line. Thirdly, someone may attempt to break into the line itself or equipment located in the telecom well to steal the transmitted information. At present, telecom companies work mainly on the principle of solving problems after they have appeared. If the line has stopped working, then workers search “locally” for a break or too much attenuation with the help of common OTDR manually and specifically inserted in a few inspection wells. After that, the repair team goes to the place to fix the problems. This allows us to fix the damage quickly enough, and data transmission, temporarily not possible over the damaged line, can usually be carried out over parallel lines. However, there are important sections or private networks in which there is no possibility of duplication. Moreover, this approach does not allow for protection from intrusion/theft of the associated equipment in telecom wells and copper cables laid nearby, which are still quite a lot despite the constant development of fiber networks. All this causes malfunctions and unnecessary costs for telecom companies, which can be reduced by using real-time monitoring systems based on a phi-OTDR. The phase-sensitive reflectometer will allow online monitoring of the state of the telecommunication line, detecting external influences on wells and unauthorized manipulations of them without opening or penetrating into the well. Recognition of pertuative events is possible with high accuracy by using neural network algorithms. This will prevent disruptions in the fiber communication line by anticipating and preventing their causes, and it will reduce repair or replacement costs.

Distributed fiber sensors were first mentioned in the early 1990s, mainly as a means of perimeter protection and intrusion control. The foundation for these research activities was laid by Taylor’s group [16,17]. Due to imperfections in technologies and components, the research continued for more than 10 years in terms of the general principle of the system and experiments in laboratories and landfills. Implementations on real physical infrastructures began only in the last 15 years. However, the large structures monitored were not communication lines but pipelines and roads [18,19,20,21]. In the last few years, there have been works on the introduction of optical reflectometers with sensors based on weak Fiber Bragg Gratings (FBGs), which allow for increasing the sensitivity of the system and obtaining data with less noise [22,23,24,25,26,27,28,29].

To date, one of the most promising ways of further developing phi-OTDR is through the evolution of signal processing algorithms. The key factor nowadays is providing the ability to recognize different types of impacts in addition to their detection and high-precision localization. Correct and specific signal recognition is particularly important for solving the tasks of monitoring the urban telecommunications network and protecting telecommunication wells. It allows us to identify the current ongoing situation and the type of intrusions: distinguish between planned work on the line or unauthorized intrusion; prevent damage that can potentially be caused by large or even small digging machines or even burglars.

In this paper, we discuss the use of a phi-OTDR for monitoring the urban telecommunications network. Its fiber cables are laid in pipes buried underground. Periodic access is possible through telecommunication wells located in the city at different points: streets, courtyards, and parks. The main goal is to test the possibility of using a phi-OTDR as a monitoring system that reports the fact of opening a well and penetration into the well. The purpose of the invasion was not necessarily limited to the fiber network. The paper will present the principle of operation of the optical measurement system and its detection algorithms, providing the features of its application for a fiber telecom network. An effective alarm model, tailored to the specific application, is demonstrated as applied to experimental data; therefore, a comparative analysis of various methods of intrusion detection is performed.

## 2. Theory

### 2.1. The Phi-OTDR Operation Principle

A conventional optical reflectometer is widely used in the fiber-optic communication industry. It can detect significant violations of the fiber line’s integrity and location. However, it can just report the overall condition and parameters of fibers and connections [30], detecting the damage to the communication line only as a fait accompli. In contrast, a phi-OTDR can provide real-time information about the presence of external acoustic and vibration influences on a fiber line along its entire length, with the ability to detect the specific location of the disturbance as well as different possible causes of the influence on the optical fiber stub. Since all intrusions into telecommunication wells are accompanied by a certain set of acoustic and, in general, vibration signals, this makes it possible to use phi-OTDR as a monitoring system. It will be able to notify people in advance about the ongoing possibility of damage to the fiber line or neighboring cables, even at the stage of the beginning of penetration or just when heavy machinery is approaching the fiber track. Additionally, a classification of different acoustic signal sources is possible depending on the nature of their action/impact on the surrounding fiber cable.

**Figure 1:**
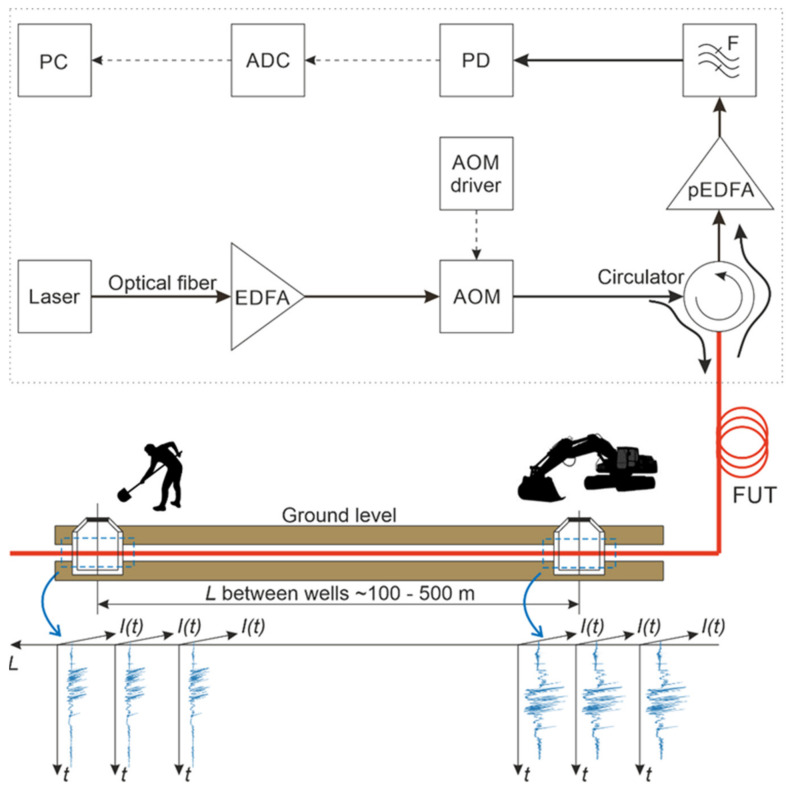
Scheme of a sensor based on phi-OTDR.

Figure 1 shows a phi-OTDR scheme and a stylized, simplified representation of the sensor cable located between two consecutive wells. Its main components and working principles are as follows: The radiation from a laser source (a semiconductor laser with external cavity stabilization, linewidth < 1.5 kHz, power 10 mW) goes to an EDFA booster to increase the power level to 250 mW. The level is lower than a nonlinear effect threshold but is still enough for signal detection from the far end of the sensing fiber. Next, the acousto-optic modulator (AOM) generates time pulses with a duration of 200 ns, which are sent through the circulator in the line of fiber under test (FUT). The pulse repetition rate was *f_rep_* = 1 kHz and was limited by our setup equipment. A theoretical limit can be calculated from FUT length as follows: *l_FUT_*: *f_MAX_* ≤ 2*nl_FUT_*/c (c—speed of light in vacuum, *n*—refractive index of the fiber core*).* The FUT is located in a cable that runs from the main communications hub through a sequential set of pipes and telecommunication wells throughout the city. Passing through the sensing fiber, light waves scatter back because of scattering centers, which are natural core refractive index inhomogeneities. The backscattered waves interfere with each other within each section of the line, with a spatial resolution determined by the half-width of the optical pulse duration *τ_p_*: *Δz =* c*τ_p_/(2n).* So we obtain high and low-intensity values from each spatial coordinate of the FUT. These values remain stable until there are no fiber vibrations from intrusion, so scattering center coordinates are practically constant. Through a circulator, the backscattered optical signal goes to the receiver unit, which consists of an erbium-doped fiber preamplifier (pEDFA), a narrow bandpass optical filter (F), a photodetector (PD), an ADC, and a PC. The pEDFA increases the signal level before optical detection, operating at a constant output power regime with an output signal level of 5 dBm. Then, pEDFA’s spontaneous radiation is suppressed by the F with a bandpass of 0.3 nm and a central wavelength similar to the laser wavelength. Finally, the signal is sent to a PD. The voltage in its output is digitized by the ADC and processed on the PC. The ADC has a sampling frequency of *ν*_ADC_ = 50 MHz, which is equivalent to a 2 m spatial digitizing step according to the formula: *Δz_ADC_* = c*/(2nν*_ADC_*)*. As a result of the interference of backscattered waves with random phases, rugged reflectograms are formed. They provide the dependence of the recorded signal intensity on time for each point of the line. Schematic examples of time sequences are shown in Figure 1. A reflectogram sequence where each one is retrieved from different backscattered light pulses is also called a ‘waterfall’. It is a three-dimensional representation of the signal in the output of the phi-OTDR, which shows an array of all the reflectogram variations between different pulses of AOM. With this sensor, one can obtain real-time information about the vibration state of each point of the optical cable as well as its surroundings by analyzing the ‘waterfall’. The ‘waterfall’ is continuously provided on the PC, so we can obtain information about the frequency and intensity of disturbance acoustic effects along the entire optical fiber length.

### 2.2. Features of the Sensing Line

As noted above, the sensor fiber is located in a cable that runs from the main communication node through a sequential set of pipes and telecommunication wells around the city. From some of the wells, there are branches into buildings for consumers. The length of the line from the measurement input node to the far end of the distributed sensor could reach more than 30 km. The main difference between this application and the monitoring of pipelines and perimeters is that telecommunication wells are vulnerable in urban conditions. Damage to the cable in the pipe can occur in exceptional cases. Instead, the unauthorized opening of a telecommunications well in order to damage a fiber line or steal copper cables is one of the most frequent violations.

For the above reasons, continuous processing of each sensor point along the fiber is not required. It is only necessary to control segments about 20 m long, corresponding to the positions of the wells and a relatively short fiber length in their proximity. This distance includes seasonal adjustments for cold and hot seasons resulting from thermal changes in cable length. Despite the fact that there are a huge number of such wells in the city and the distance between the wells can vary from 50 to 300 m, fiber sensing systems in any case require mandatory binding on the map. The binding is carried out only once during installation. This feature significantly reduces the resources needed for signal processing, so that in telecommunication network wells monitoring, the processing is required for at least five times fewer sections with the same line length in comparison to full-length pipeline monitoring applications. This allows you to use more resource-intensive algorithms while continuing to execute them in real-time.

### 2.3. Overview of Recognition Methods for Phi-OTDR Signals

Publications in the field of phi-OTDR signal recognition have been appearing since 2010 [31,32,33,34]. The main methods used can be divided into two groups. The first group involves a transition from spatial characteristics to frequency characteristics, followed by an analysis of the resulting distributions in the frequency domain. In general, this transition is carried out using the Fourier transform or the wavelet transform. Methods for using the wavelet transform are described in [35,36]. In [35], the authors apply a packet wavelet transform to localize disturbances near the sensor cable. A conversion is constructed for the signal from each sensor coordinate, and then the relative energy is calculated. If the energy at any point in time exceeds the specified threshold, then a disturbance has occurred. This approach made it possible to detect disturbances with an accuracy of up to 150 m and a false alarm rate of 2%. The authors [36] compare the efficiency of the wavelet transform and the packet wavelet transform for the task of identifying three types of events: background noise, artificial earthworks, and vehicle passage. Due to the fact that different events have different frequency content characteristics, an assumption was made about the possible efficiency of using energy distributions to identify and classify different types of events. The distributions of wavelet energies (WE) and packet wavelet energies (WPE) were used as features for the description of events. An artificial neural network with two hidden layers was chosen as a classification algorithm. The percentage of correct detections when using WE was 91.1%, while when using WPE it was 94.4%.

The second group of methods analyzes raw signals without preprocessing. The most striking examples are [37,38,39]. In the article [37], the authors propose to analyze signals and classify them based on morphological features. They emphasize that traditional methods based on Fourier or wavelet transformations require a lot of time, especially in situations where several events occur simultaneously. The idea of the article is to analyze a two-dimensional time–space signal (a waterfall) as an image. To localize events, the Otsu method [40] is used to search for the binarization threshold. Next, each selected object is assigned a set of characteristics describing the event area. The full list can be found in the article. The proportion of correctly recognized events reaches 98%.

Neural networks have found wide applications in the field of signal analysis. A.V. Makarenko [38] uses an ensemble of convolutional neural networks for automatic feature extraction and subsequent classification. The accuracy of the algorithm reaches 91%. In [39], the authors present the original signal as a three-channel image. The channels correspond to the real part, the imaginary part, and the values of the Fourier transform coefficients. The classical convolutional neural network architecture is used to work with images, consisting of a sequential alternation of convolutional layers, max pooling layers, and nonlinearity. On six types of events, the average accuracy is 95%. In [41], the authors investigate the dependence of the quality of a one-dimensional convolutional network on the number of layers in it.

Large amounts of data that have to be processed and analyzed when using DAS often require powerful computing facilities and expensive servers. In order to optimize computing power, a number of works aim to create algorithms that would make it possible to perform the required calculations by spending the least amount of resources [42].

Our goal in this work was to choose an algorithm that would provide good recognition quality and the ability to monitor a large number of wells in the city online.

## 3. Experiment

### Description of Data Collection. Formation of an Array for Analysis

**Figure 2:**
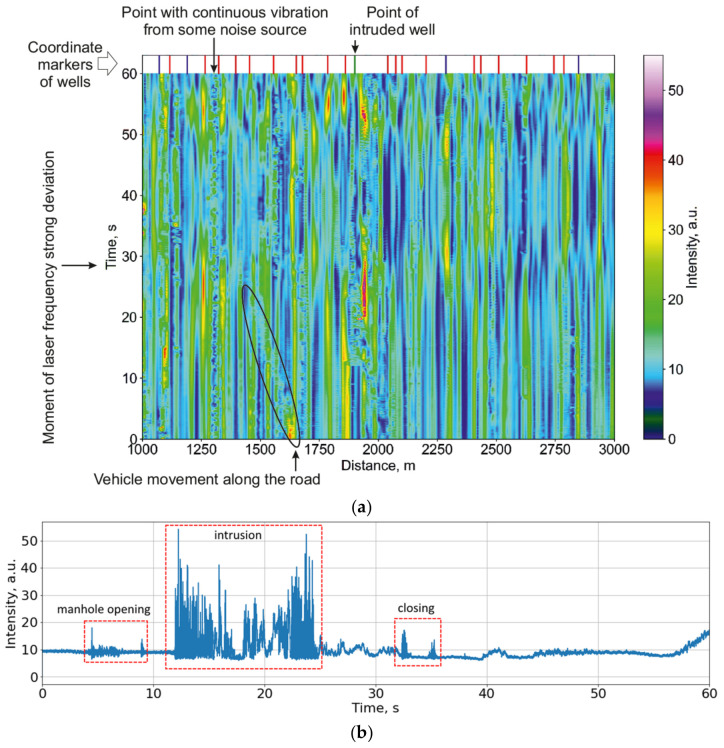

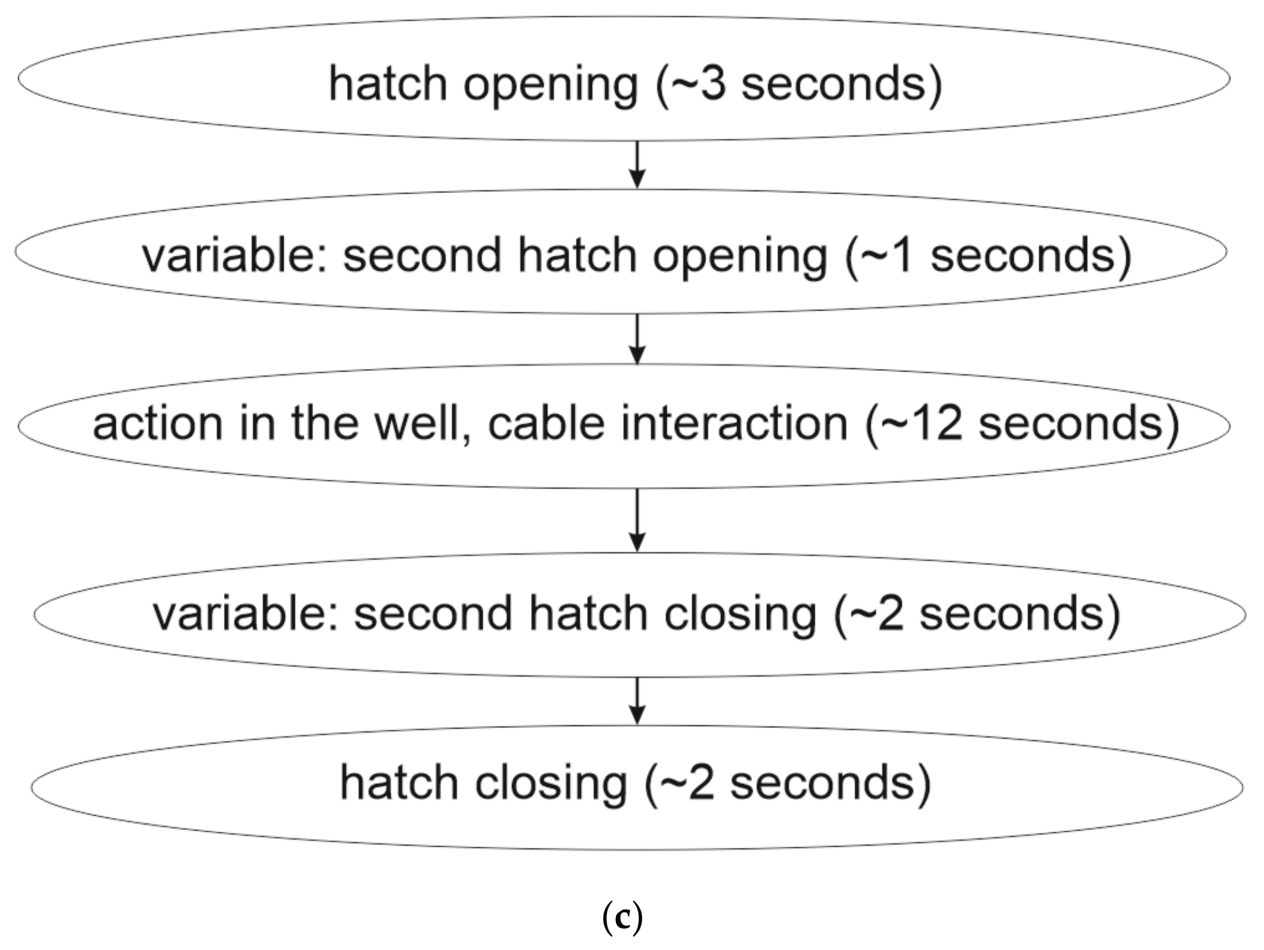
Examples of actions with fiber sensors. (**a**) Different environmental noises and signals on the phi-OTDR waterfall plot; (**b**) signal evolution in time during intrusion into a manhole; (**c**) common scheme of intrusion events.

The scheme of the phi-OTDR used in our experimental studies is shown in Figure 1. The sensor part was realized based on cables that were part of the urban fiber-optic infrastructure, with the ability to access them through communication wells. During experiments, these wells were intruded, and the corresponding phi-OTDR signals were collected. Based on the analysis of these signals, algorithms for intrusion detection and disturbance types’ determination were designed. An example of the reflectogram obtained from the experiment as well as a measured waterfall are shown, respectively, in Figure 2a,b.

**Figure 3:**
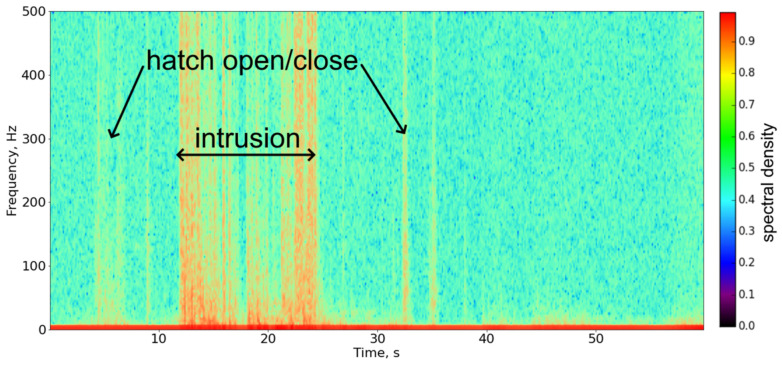
Example of a signal spectrogram. It can be seen that the effect of the disturbance is manifested in the entire spectral range recorded by the device. The main frequencies are in the range from 15 to 70 Hz.

Each telecommunication well had one heavy metal cover and an average depth of 1 to 4 m. In order to carry out the descent into the well, a portable ladder was used. In practice, the use of such equipment inevitably leads to direct interaction with the equipment and cables inside. Moreover, due to the large number of cables in a small space, it practically does not matter which of them will be affected: the fiber-optic sensor cable or the neighboring copper one. In any case, the signal will be of high intensity due to the contact between the cables. The frequency of the disturbance impact may vary depending on human actions, but in intensity phi-OTDR, this frequency can usually be observed in the entire recorded range up to 500 Hz. An example is shown on the spectrogram in Figure 3.

A series of experiments on 51 different wells on one fiber line were carried out according to the scheme shown in Figure 2c. Examples of the signal evolution at one coordinate point during different operations in the well are shown in the waterfall diagram in Figure 2a. The coordinates of each well in the sensor line were determined in the first experiment via standard-imposed periodic impacts in proximity to the fiber cable.

**Figure 4:**
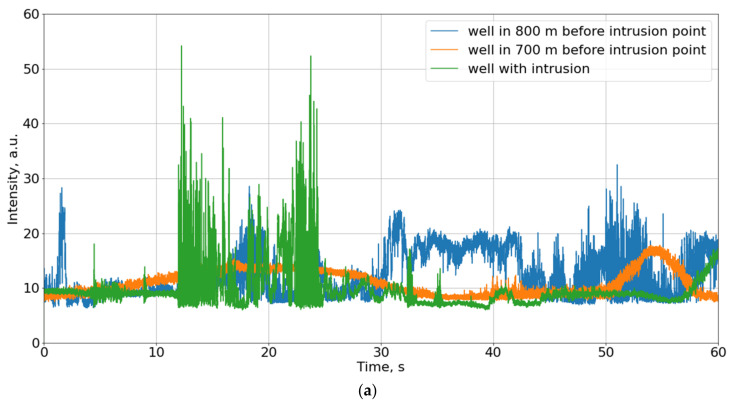

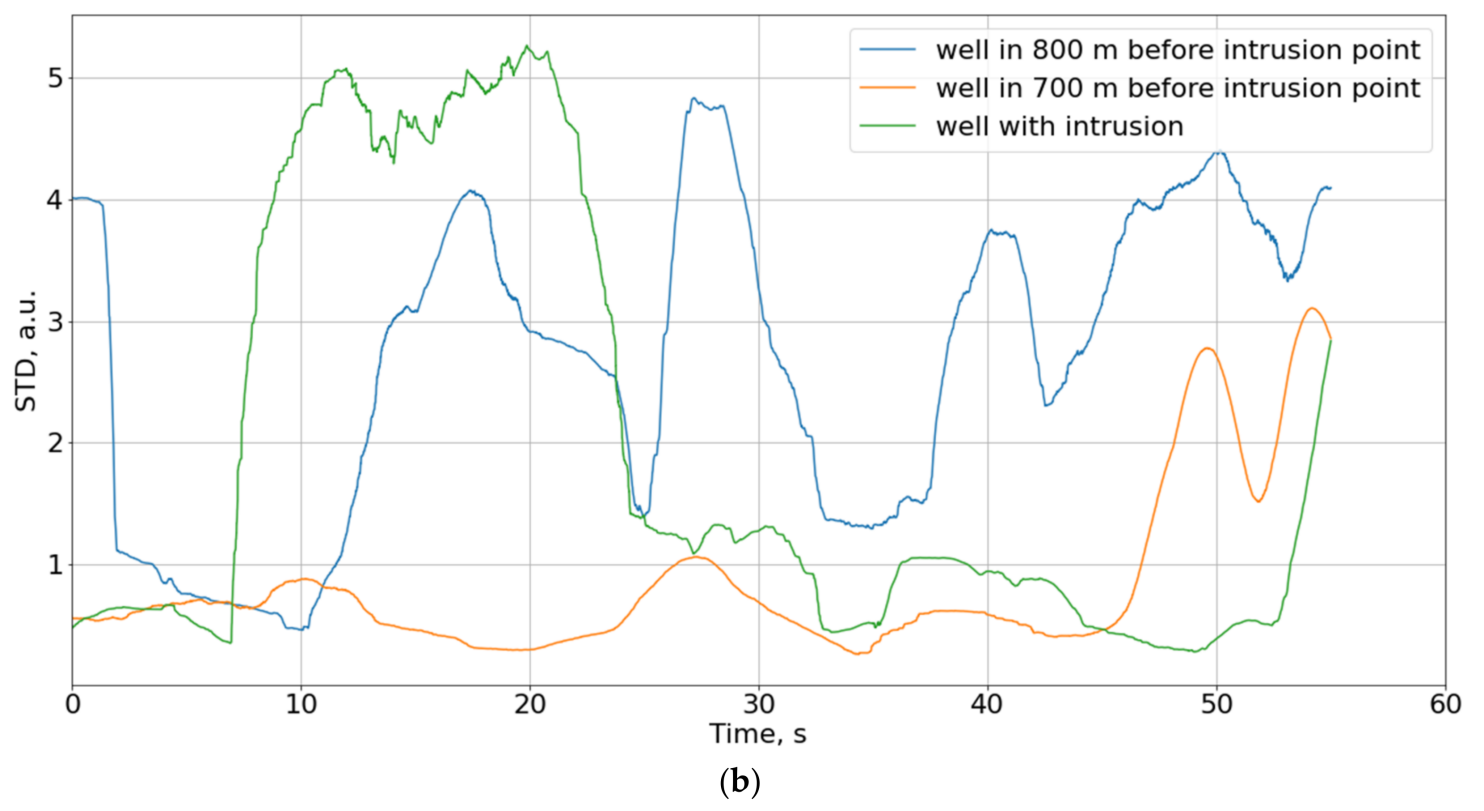
Examples of signals in different wells: green line according to wells with intrusion, environmental, and system noises; blue and orange curves according to wells with no intrusion, just environmental and system noises: (**a**) intensity on the photodiode without any processing; (**b**) standard deviation of intensity on the photodiode in a sliding 5-s window.

The main difficulties in analyzing incoming signals are now highlighted. Firstly, the urban infrastructure creates a lot of noise. Well covers are often located on footpaths or roadways, thus collecting many different vibrations even under normal operating conditions. The effects of a person’s steps on the well, or the passage of transport, can be easily confused with the opening process. A lot of traffic can be detected as an impact on the cable. Secondly, noise also appears due to the instability of the system components (the noise of the optical preamplifier and the laser frequency drift). The first component has an approximately constant level if measured in short-term sections. However, the second component may manifest itself in different ways, unlike the result of random interference. If it generates the minimum interference, the sensitivity in this area will be minimal, and the signal amplitude will change little even with strong disturbance exposure. When reaching the interference maximum, even in the absence of exposure, the signal fluctuations will have a large amplitude, and this can be taken as an intrusion in the well. Examples are shown in Figure 4a. All the signals have noise because of the instability of the system components. Nevertheless, their general behavior can be considered. A signal from the fiber near the well-being disturbed by the intrusion is shown in green, containing a slowly varying component when there is no intrusion. When the well is disturbed, sharp and intense signal variations appear between 10 and 15 s, so the intrusion can be detected. The orange curve demonstrates a good example of a signal without intrusions, as it is calm and changes slightly. A blue one relates to a well located 800 m before the intrusion point. Despite the fact that this well is only affected by environmental and system noise without intrusions, the signal can be confused to contain information about someone operating with the well.

Figure 4b shows the standard deviation of intensity on the photodiode in a sliding 5-s window, the same as the sample size. Analyzing standard deviation changes in time helps both to eliminate noise and to distinguish signal areas with high intensity. However, these curves also do not allow distinguishing wells with and without intrusions.

Methods based on only amplitude or standard deviation tracking will not present information with a good quality of recognition because, in some cases (see blue line in Figure 4), we will observe high amplitude or/and standard deviation when there is no intrusion, just environmental and system noises. In order to eliminate uncertainties, it is necessary to recognize the signals.

**Figure 5:**
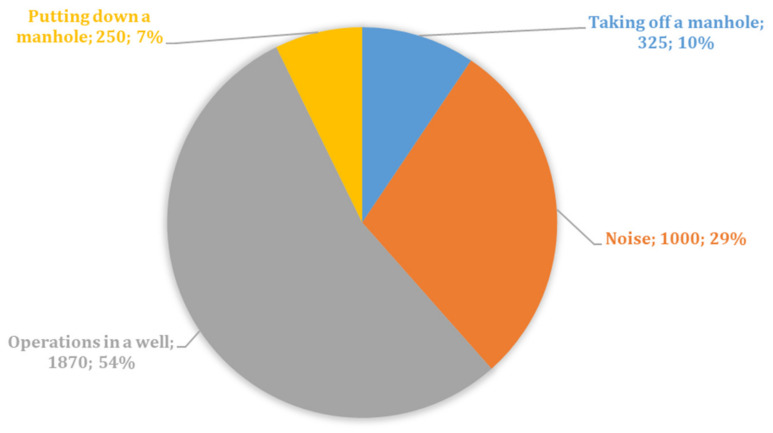
Histogram of the number of event types.

Further, time dependencies were taken from the selected waterfall sections. Three types of events were manually marked on them: the opening of the hatch, the action in the well, and the closing of the hatch. An array of intensity values for 5 s was assigned to each event. We called such an array an object. To increase the sample size of the data, we also took data from the two previous and two following coordinate points. This makes it possible to partially compensate for the low sensitivity of the sensor in the case of the interference minima described above or to process a stage with a lower noise level in the case of a maximum. In addition, from sensor coordinates located outside the well area, objects were selected as the noise samples. This choice allowed for additional options for the noise environment created by the urban infrastructure and the completeness of all signs of interference. In total, the study included a sample of 3445 different objects. The distribution of events in the resulting sample can be seen in Figure 5. The dataset was divided into training and test parts in a ratio of 4:1. The selection of hyperparameters was carried out based on cross-validation of the training sample at 𝑘 = 5. As the main criterion for the quality of the algorithm, we consider the proportion of correct answers—accuracy.

In this study, the recognition of four object types from the original sample was carried out. This problem can be reduced to the problem of classification in the feature space. Therefore, it is possible to distinguish the following stages in solving the problem:

- construction of feature spaces for describing objects;
- application of various classification methods and algorithms;
- comparison of the results of the algorithms.

For the final comparison, three characteristics were used:

- signal spectral density (SD),
- logarithmic filter bank energy (FBE),
- wavelet packet energy (WPE),

The methods and algorithms used were:

- Logistic regression [43],
- Random Forest [44],
- Gradient boosting [45].

A convolutional neural network (CNN) was also used, with three convolutional and one fully connected layer; its structure is presented in Listing 1.

**Listing 1.** CNN structure used for signal classification.1. sig_inp = Input(shape=(4990,1)) # input signal2. conv1 = Conv1D(16, 20, activation=“relu”)(sig_inp)3. pool1 = MaxPooling1D(pool_size=3)(conv1)4. conv2 = Conv1D(16, 10, activation=“relu”)(pool1)5. pool2 = MaxPooling1D(pool_size=3)(conv2)6. conv3 = Conv1D(16, 10, activation=“relu”)(pool2)7. pool3 = MaxPooling1D(pool_size=3)(conv3)8. flat = Flatten()(pool3)9. dense1 = Dense(64, activation=“relu”)(flat)10. output = Dense(4, activation=“softmax”)(dense1) # output class

To confirm the effectiveness of this architecture, several experiments were conducted in which the quality of different depth models and architectures was compared. The training was carried out by minimizing the functional loss for several classes. The stochastic gradient descent method was used to find the minimum [46].

## 4. Analysis

For the experiments, the implementations of logistic regression and random forest were taken from the scikit-learn library [47], and gradient boosting implementations from the XGBoost library [48], Keras [49], and TensorFlow [50] libraries were also used.

The results of all algorithms can be seen in Table 1. The quality assessment by the accuracy metric was carried out on the same hold-out set.

As can be seen from the table, SD is generally performing better than WPE and FBE, with the last one being generally, except with logistic regression, the least performing of all. The best quality among the models based on frequency features was shown by gradient boosting. It is also worth noting that logistic regression works much better with SD feature space than WPE and FBE. It can be assumed that in the other two spaces, there are non-linear dependencies that the linear model is unable to restore. In addition, it is worth paying attention to the fact that the quality of the random forest is lower than the quality of the logistic regression on SD features. This feature can be explained by the high dimension of the SD space (*d* = 2500 for 5 s of data with 1 kHz discretization). Since the tree-building algorithm is greedy, finding the optimal partition for a large number of features becomes much more difficult. In addition, it is worth noting that all three algorithms showed the maximum quality when using features that describe the signal spectral density. Among all models, the convolutional neural network showed the best quality.

**Figure 6:**
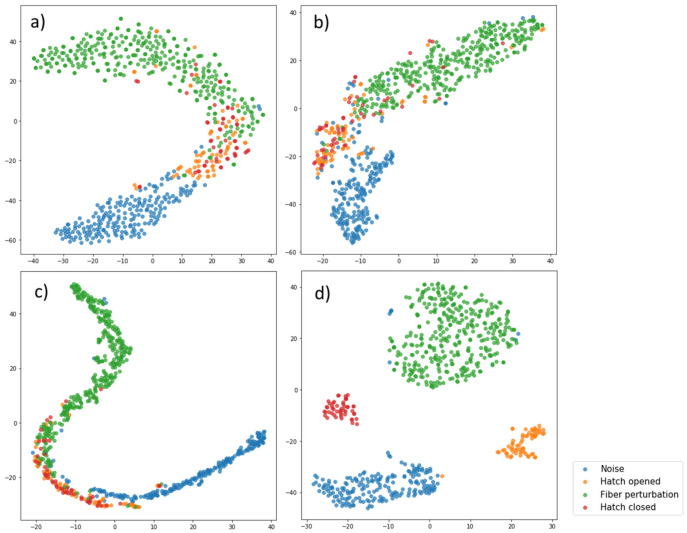
Visualization representation of different feature spaces: (**a**) SD, (**b**) FBE, (**c**) WPE, and (**d**) values of activations on the penultimate layer.

Figure 6 shows visualizations of different feature spaces. For the neural network, the values of activations on the penultimate layer were taken. Since it is impossible to directly depict a multidimensional space, the method of non-linear dimensionality reduction (t-SNE) was applied [51]. This method consists of two steps. First, a probability distribution is created in a high-dimensional space such that close objects have a high joint probability, while different objects have a lower joint probability. In the second step, a similar probability distribution over points in a space of lower dimension is specified, and the Kullback–Leibler divergence [52] between the resulting distributions is minimized. For visualization convenience, the dimension of each feature space was reduced from the original to 𝑑𝑖𝑚 = 2 in Figure 6.

It can be seen that the most distinguishable event classes are shown in Figure 6d. These results are quite consistent with the quality of the constructed models. It is worth noting that a large number of events, such as “taking off a manhole” and “putting down a manhole”, lie together. This observation is very intuitive because similar manipulations are actually performed in the two cases and should create interference of a similar nature. In all four cases, events of the types “noise” and “operations in the well” are best separated. This property will be useful in practice since one of the main tasks of the algorithm is to determine the intrusion and violation of the cable state.

In addition, we analyze confusion matrices [53]. Table 2, Table 3, Table 4 and Table 5 present confusion matrices for WPE-based logistic regression, WPE-based gradient boosting, SD-based gradient boosting, and CNN, respectively. The first three cases give us the possibility to understand which situations generate the main problems for classifiers and guess the reasons. The last case shows the accuracy and outliers of the best-tested classifier.

Based on the confusion matrices, the following conclusions can be drawn: Logistic regression gives poor results when allocating the “hatch opening” and “hatch closing” classes, while gradient boosting has shown good results. These logistic regression indicators may mean that the features contain nonlinear dependencies, which implies that there is no good way to separate hyperplanes. Gradient boosting, due to its nature, is able to find nonlinear separating surfaces and capture nonlinear dependencies. Most often, the closing of the hatch was mistaken for an opening, and perhaps this is because the two actions performed have a similar nature and have a similar effect on the sensor cable.

The entanglement matrices for the two most accurate algorithms, namely gradient boosting with SD features and convolutional neural networks, show that both algorithms well separate noise events from the combined group of non-noise ones. This indicator is very important from a practical point of view. Using the apparatus of mathematical statistics, we can set the null hypothesis 𝐻0, corresponding to the absence of any intrusion, and the alternative hypothesis 𝐻1, corresponding to the intrusion. Then, based on practical application, it is more important for us to minimize the number of errors of the second kind responsible for the incorrect acceptance of the null hypothesis: mistaken detection of no intrusion, when actually one intrusion has occurred, is the misdetection that needs to be minimized.

## 5. Conclusions

In this paper, a fiber optic sensor system based on phi-OTDR for manhole integrity monitoring is presented. The main stages of intrusion and their characteristics were described. Two noise sources were highlighted and shown in the collected data with a description of their nature. Three different methods of constructing feature spaces were proposed for the events occurring in fiber-optic telecommunication network wells monitoring. These methods are based on the spectral density of the signal, the logarithms of the filter energies, and the energies of the packet wavelet transform. During the comparison of four machine learning methods, it was found that the convolutional neural network shows the maximum quality according to the metric accuracy of detection, with an indicator of 98.6%. It was reached with the CNN configuration with three convolutional and one fully connected layer. Monitoring systems with the proposed algorithm can provide continuous monitoring of urban telecom lines and prevent their corruption.

## Figures and Tables

**Table 1:** Processing results for chosen classifiers.

Classifier	Accuracy, %		
	Used Features		
	SD	FBE	WPE
Logistic regression	95.65	84.33	82.29
Random forest	92.31	90.42	92.02
Gradient boosting	96.23	90.71	93.61
Convolutional network	98.55		

**Table 2:** Confusion matrix for logistic regression with WPE features.

True Class	Predicted Class			
	Noise	Taking Off a Manhole	Operations in the Well	Putting Down a Manhole
Noise	207	1	7	0
Taking off a manhole	32	14	21	0
Operations in the well	9	0	346	0
Putting down a manhole	18	17	17	0

**Table 3:** Confusion matrix for Gradient boosting with WPE features.

True Class	Predicted Class			
	Noise	Taking Off a Manhole	Operations in the Well	Putting Down a Manhole
Noise	208	1	6	0
Taking off a manhole	0	53	11	3
Operations in the well	2	2	348	3
Putting down a manhole	2	9	5	36

**Table 4:** Confusion matrix for Gradient boosting with SD features.

True Class	Predicted Class			
	Noise	Taking Off a Manhole	Operations in the Well	Putting Down a Manhole
Noise	193	0	7	0
Taking off a manhole	1	62	2	0
Operations in the well	0	7	365	2
Putting down a manhole	4	2	4	40

**Table 5:** Confusion matrix for CNN results.

True Class	Predicted Class			
	Noise	Taking Off a Manhole	Operations in the Well	Putting Down a Manhole
Noise	191	0	8	1
Taking off a manhole	0	65	0	0
Operations in the well	0	0	374	0
Putting down a manhole	0	0	1	49

## Data Availability

The data presented in this study are available on request from the corresponding author.
